# Endoscopic Detorsion Results in Sigmoid Volvulus: Single-Center Experience

**DOI:** 10.1155/2020/1473580

**Published:** 2020-05-13

**Authors:** Necattin Firat, Baris Mantoglu, Kayhan Ozdemir, Ali Muhtaroglu, Emrah Akin, Fehmi Celebi, Altintoprak Fatih

**Affiliations:** ^1^Sakarya University Medical Faculty, General Surgery Department, Adapazarı, Turkey; ^2^Sakarya University Educational and Research Hospital, General Surgery Department, Adapazarı, Turkey

## Abstract

Endoscopic detorsion is the first-line recommended treatment modality in sigmoid volvulus patients who have no peritoneal irritation signs on admission. In this paper, we present the results of endoscopic detorsion procedures applied at the time of presentation with the diagnosis of sigmoid volvulus and review the current literature about this topic.

## 1. Introduction

Although volvulus can occur in various localizations in the gastrointestinal tract, they are most frequently (65–80%) seen in the sigmoid colon because of the anatomical features that facilitate the rotation of the mesentery [1]. Sigmoid volvulus is included in intra-abdominal emergency surgical pathologies, and delayed diagnosis increases the risk of developing complications such as intestinal ischemia, necrosis, and perforation [2]. Today, thanks to the development of the possibilities of emergency services and the presence of imaging methods that can be completed very quickly, especially as a consequence of advances in radiological examinations, the diagnosis of sigmoid volvulus can be established immediately and treatment planning can be made [3].

Endoscopic intervention opportunities and diversity have progressed rapidly in the last two decades all over the world, and many clinical conditions, which reported to be treated with surgical intervention in the past, have now become intervenable without the need for surgical intervention with endoscopic methods [4]. The same is valid for sigmoid volvulus, and in case of early diagnosis when complications requiring surgical intervention are not developed, endoscopic detorsion becomes the first recommended treatment method in the presence of sigmoid volvulus [3, 5].

In this text, the results of patients who have undergone endoscopic detorsion with the diagnosis of sigmoid volvulus are given, and the current literature on this subject is reviewed.

## 2. Materials and Methods

The records of patients who were admitted to the Sakarya University Faculty of Medicine Emergency Department with complaints of abdominal pain, gas-stool evacuation failure, and/or vomiting between January 2013 and January 2019 and who were diagnosed with volvulus in any localization in the gastrointestinal tract after clinical and radiological examinations were retrospectively reviewed. Volvulus patients who had volvulus detected with signs outside the sigmoid colon and who underwent surgical treatment under emergency conditions due to signs of peritoneal irritation at the time of admission were excluded from the examination. The records of the patients who were diagnosed with sigmoid volvulus after the first application to the emergency service and who had undergone endoscopic detorsion evaluated in terms of demographic data, procedure time, hospitalization time, morbidity-mortality rates, and early and late recurrence rates were evaluated. The data were evaluated using the appropriate statistical methods (mean ± standard deviation and percent (%)).

In our clinic, we accept the following parameters in the decision to apply endoscopic detorsion:

- 〉18 years of age.
- No signs of peritoneal irritation on physical examination at the time of application.
- Findings compatible with sigmoid volvulus in radiological examinations performed at the time of application (conventional abdominal X-ray and abdominal computed tomography examination).
- No findings of complications (such as intra-abdominal free air, signs of ischemia in the colon wall, and intra-abdominal stool contamination) in the same radiological examinations.
- Neither shock and/nor sepsis findings (such as hypotension, tachycardia, and fever).

Bowel preparation was not performed before the procedure, and all procedures were completed by experienced staff working in the surgical endoscopy unit within 1 hour after diagnosis. All procedures were performed with flexible endoscopy compatible with the Fujinon processor VP-4450HD device in the left lateral decubitus position after sedation anesthesia was applied. During the endoscopic procedure, the image of the vortex was viewed in the torsioned colon, and the endoscope was advanced by targeting the most extreme point of it. During the progression, the procedure was terminated to prevent perforation by examining the intestinal wall as ischemic; furthermore, the patients were delivered to the operating room for emergency surgery. In patients with no evidence of ischemia in the intestinal wall, detorsion was performed without any difficulty, and the air was aspirated until the solid stool was noticed. Rectal tubes were not applied to the patients after the procedure.

## 3. Results

**Figure 1:**
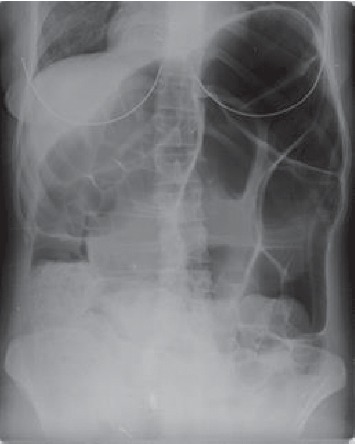
Sigmoid volvulus X-ray.

**Figure 2:**
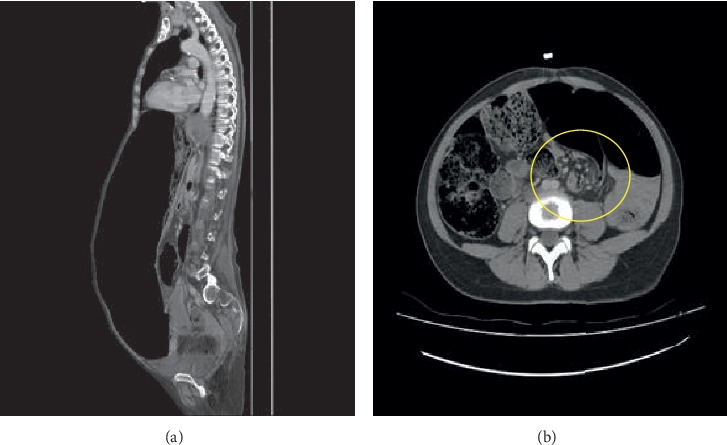
(a) Sigmoid volvulus abdomen tomography sagittal plane. (b) Sigmoid volvulus hurricane sign.

Between the specified dates, 36 patients were diagnosed with volvulus in the gastrointestinal tract. As a final diagnosis, three patients had gastric volvulus, and three patients had cecum volvulus, while the remaining 30 patients were diagnosed with sigmoid colon volvulus (Figures 1-2). Surgical treatment was applied to patients diagnosed with stomach volvulus and cecum volvulus under emergency conditions.

In ten (33.3%) of the patients diagnosed with sigmoid colon volvulus, surgical treatment was performed in emergency conditions without endoscopic detorsion due to signs of peritoneal irritation at the time of admission. Eight (80%) of these patients underwent Hartmann procedure, while two (20%) of the patients underwent detorsion and sigmoidopexy surgery.

**Figure 3:**
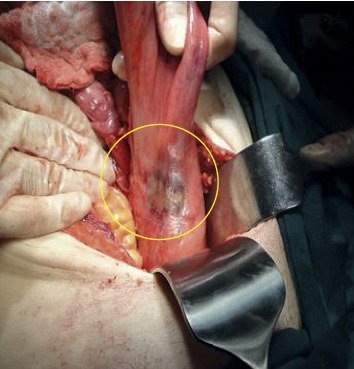
Local ischemia.

**Figure 4:**
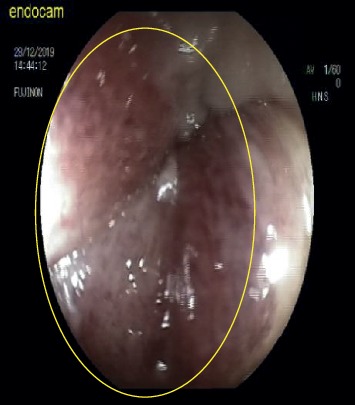
Ischemic mucosal findings.

Endoscopic detorsion was performed in 20 patients diagnosed with sigmoid colon volvulus. Of these patients, ten were female (50%), ten were male (50%), and the mean age was 61.8 ± 23.5 (mean ± standard deviation) (min.21, max.95 y). Five patients had neurological or psychiatric disorders, three had cardiovascular disease, and two had previous abdominal operations. The time between the onset of abdominal pain complaints and admission to the hospital ranged from 6 to 18 hours. Though detorsion was completed without complications in eighteen (Table 1 demographic data) (90%) patients, detorsion was unsuccessful in two patients (10%) and surgical treatment was performed (Hartmann procedure). In two (10%) of the patients with successful detorsion, an emergency surgical intervention was decided after the procedure due to local mucosal ischemia findings identified in the localization corresponding to the area where the volvulus transpired in the endoscopic examination (Figures 3-4).

Following the detorsion procedure, all patients were recommended with elective surgery, but only three patients (15%) accepted the surgery and these patients underwent laparoscopic sigmoid colon resection under elective conditions. Volvulus occurred in five (25%) of 13 patients (65%) who did not accept the surgery and was followed up clinically within the first year (in the first month in 4 patients and in the 9th month in one patient). However, three (15%) of the patients who developed recurrence had undergone surgical intervention in emergency conditions due to the presence of peritoneal irritation findings and two (10%) of the patients underwent re-endoscopic detorsion procedure; afterward, these patients underwent a laparoscopic resection anastomosis and a sigmoidopexy operation electively (Table 2). After endoscopic detorsion, one patient had died due to cardiovascular disease, and seven patients who were followed for a mean of 15 months (8–30) did not develop recurrence.

## 4. Discussion

The coexistence of sigmoid volvulus, which is frequently encountered at older ages, with chronic neuropsychiatric disorders, immobility, and chronic constipation, is frequently emphasized [6–8]. While researching gender differences in the literature, studies are reporting male domination as well as there are studies indicating that there is no gender difference [9–11]. It has been stated in studies that there is male gender dominance because the sigmoid colon mesentery length is higher in males [12–14]. No gender difference was observed in our study, and the results were consistent with the literature data in terms of comorbid diseases accompanying demographic data.

Neuropsychiatric diseases, which are present in some of the patients who develop sigmoid volvulus, cause late detection of the clinical symptoms because they make it difficult for patients to express themselves, which may result in ischemia, perforation, peritonitis, and sepsis. 25% of our patients had neuropsychiatric disorders. The fact that the timing of admission to the hospital in sigmoid volvulus in the literature is one of the most effective parameters on morbidity and mortality rates supports this fact [15, 16]. The time of admission to the hospital is also an important parameter in determining the type of treatment to be applied, and the success chance of minimally invasive procedures decreases or does not remain at all, after the ischemia development [15]. In our study, the duration of admission obtained to be 14 to 18 hours in patients with mucosal ischemia identified during endoscopic examination.

Critical points in the treatment of sigmoid volvulus are detorsion of the volvulus segment and the prevention of recurrence. Although endoscopic methods and surgical methods outlined the treatment options, there were some differences of opinion about their priorities in the past. However, the idea that endoscopic methods should be applied primarily in patients without acute abdominal syndrome findings has started to gain weight and the statement that “the first treatment of sigmoid volvulus should be endoscopic detorsion in the absence of colonic ischemia or perforation” has been a common thought in the literature [8, 17–20]. It has been reported that performing detorsion using rigid or flexible endoscopes does not affect success rates, but guidance with flexible endoscopes is better tolerated by patients [8, 21–23]. In our study, all procedures were performed with a flexible endoscope, and no negativity was encountered in terms of patient tolerance. Two patients' endoscopic intervention was terminated due to mucosal ischemia (10%) and unsuccessful detorsion (10%); furthermore, those patients went to emergency surgical intervention. Intestinal ischemia was detected during operation in one of the patients whose endoscopic detorsion failed. It was noted that this patient's admission to the hospital was 14 hours after the onset of pain. We think that the risk of intestinal ischemia will increase as the duration of the torsion increases.

The primary anastomosis or Hartmann procedure after resection in the surgical treatment of sigmoid volvulus remains the most common options [8, 15, 24, 25]. Providing intestinal continuity, in other words, anastomosis after resection is a safe option in patients without gangrene [8, 12, 13, 26, 27]. Although laparoscopic approaches for emergency interventions are controversial, it has been determined that the laparoscopic procedure does not have a significant advantage over the results of sigmoid volvulus patients [28]. It stated that it would be more appropriate to perform endoscopic detorsion in the first step, instead of a laparoscopic intervention in the acute phase and to apply a laparoscopic approach within 2–5 days following detorsion [3]. Thus, it will be on the agenda that an operation that will be performed in emergency conditions can be performed safely and prepared by drawing it to an elective intervention. Today, many centers recommend elective surgery after successful endoscopic detorsion because besides being a procedure with a high success rate, endoscopic detorsion is not a definitive treatment option in most patients and high recurrence rates (30–90%) are reported [26, 29]. In our study, recurrence was observed in 5 of 13 patients who were followed up, and this rate was consistent with the literature data (38%), but we think this result may increase due to the small number of patients and short follow-up time.

As a result, endoscopic detorsion should be considered as the first treatment option in patients with sigmoid volvulus without signs of peritoneal irritation, but the elective surgery option should be put on the agenda because of the high recurrence possibility of the procedure after the emergency has resolved.

## Figures and Tables

**Table 1:** Demographic values.

		Male	Female	*n*
	Age	70.10 ± 25.00	53.40 ± 24.41	61.80 ± 23.50

Patients	Emergency intervention	4 (%20)	3 (%15)	7 (%35)
	Elective intervention	0 (%0)	5 (%25)	5 (%25)
	Followed patients	6 (%30)	2 (%10)	8 (%40)
Additional diseases	Neuropsychiatric	1 (%5)	4 (%20)	5 (%25)
	Cardiovascular	2 (%10)	1 (%5)	3 (%15)

**Table 2:** Results of endoscopic detorsion.

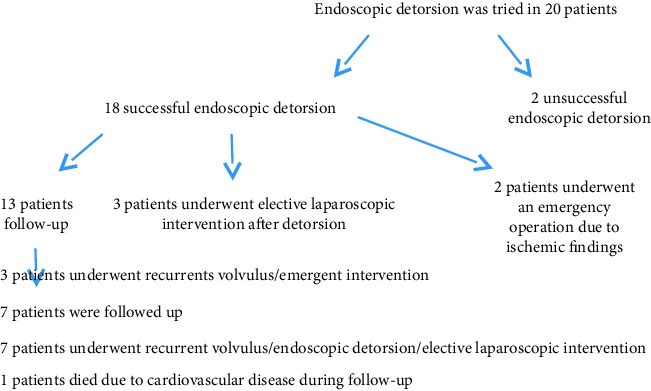

## Data Availability

There are no additional data available to share with the readers. The datasets used and/or analyzed during the current study are available from the corresponding author on reasonable request.
